# Surgical correction of ruptured aneurysms of the sinus of Valsalva using on-pump beating-heart technique

**DOI:** 10.1186/1749-8090-5-37

**Published:** 2010-05-14

**Authors:** Ansheng Mo, Hui Lin

**Affiliations:** 1 Department of Cardiothoracic Surgery, The People's Hospital of Guangxi Zhuang Autonomous Region, Nanning 530021, China

## Abstract

**Background:**

Rupture of aneurysms of the sinus of Valsalva results in abrupt onset of congestive heart failure. On-pump beating-heart surgery may reduce cardiac impairment by maintaining coronary blood flow and avoiding cardioplegia. Herein, we report the operative correction of thirty-one patients of ruptured aneurysms of the sinus of Valsalva, using the on-pump beating-heart technique.

**Methods:**

Thirty-one patients with ruptured aneurysms of the sinus of Valsalva underwent operative corrections using the on-pump beating-heart technique. In patients with fistula diameter less than 1 cm and no aortic regurgitation, the aorta was unclamped throughout cardiopulmonary bypass(CPB) while receiving antegrade heart perfusion. In remainder of patients, retrograde perfusion was used.

**Results:**

After intracardiac manipulation was complete and the nasopharyngeal temperature was raised to 36-37°C, the patients were smoothly weaned off CPB. There were no early or late postoperative deaths. All patients were in New York Heart Association functional class I at follow-up (range, 0.5-1 year). Mild-to-moderate aortic valve regurgitation was observed in one patient. No recurrence of the left-to-right shunt from ruptured aneurysms of the sinus of Valsalva was observed.

**Conclusions:**

Beating heart on pump allows adequate examination of the aortic lesion under near-physiologic conditions, allows decrease in ischemia-reperfusion injury and potentially decreases the risk of serious or fatal rhythm disturbances. On-pump beating-heart technique for repair of ruptured aneurysm of sinus of Valsalva is feasible and promising. Antegrade heart perfusion is suitable for patients with a fistula diameter <1 cm and no aortic regurgitation, and retrograde perfusion is suitable for the others.

## Background

Rupture aneurysms of the sinus of Valsalva usually results in abrupt onset of congestive heart failure. Repair of intracardiac teratosis is always performed under cardioplegic arrest. This might cause cardiac impairment leading to low postoperative cardiac output [1-12]. Therefore, myocardial protection is key to the success of these operations. Evidence from experimental and clinical work has demonstrated that on-pump beating-heart surgery reduces further cardiac impairment by maintaining coronary blood flow and avoiding cardioplegia [13-18].

Based on our previous reports [19], we have further introduced on-pump beating-heart technique for correction of ruptured aneurysms of sinus of Valsalva, and have determined the optimal heart perfusion for different situations.

## Methods

### Patients

A retrospectively review carried out of 31 consecutive patients with ruptured aneurysms of the sinus of Valsalva, who were operated upon using on-pump beating heart technique between March 1, 1993 and December 30, 2007. Preoperative variables and surgical procedures are summarized in Tables 1 and 2, respectively. The study was approved by the Institutional Human Ethics Committee, and all patients gave consent for the procedure.

**Table 1:** Patient Characteristics

Variable	N (n = 31)
Male/female	23/8
Age (years)	26.06 ± 6.71
Time interval between onset of symptoms and operation (days)	137.97 ± 159.73
Fistula	
NS-RA	4
RS-RA	9
RS-RV	18
Diameter of the fistula	
≤1 cm	28
>1 cm	3
NYHA functional class	
II	6
III	10
IV	15
Coexistent lesion	
Ventricular septal defect	9
Aortic regurgitation	7
Subaortic stenosis	1
Right ventricular outlet stenosis	1

NYHA = New York Heart Association, LV = left ventricle, RA = right atrium, RV = right ventricle, NS = noncoronary sinus of Valsalva, RS = right coronary sinus of Valsalva

**Table 2:** Procedures Performed

Variable	N(n = 31)
Surgical approach	
RA	9
RV	15
Ao	4
Ao+RV	3
Techniques for repair	
Simple closure	14
Patch closure	17
Coexistent lesion correction	
Simple closure ventricular septal defect	5
Patch closure ventricular septal defect	4
Aortic valvuloplasty	1
Aortic valve replacement	6
Right ventricular outflow construction	1
Radical resection subaortic stenosis	1
The time of cardiopulmonary bypass	60.26 ± 21.86
Method for perfusing the coronary vasculature	
Antegrade perfusion	22
Retrograde perfusion	9
The time of cross-clamping for retrograde perfusion	42.50 ± 12.47

LV = left ventricle, RA = right atrium, RV = right ventricle, Ao = aorta

### Operative procedure

Surgical repair was performed using CPB via a median sternotomy on a beating heart with mild hypothermia (32-34°C). The technique of CPB and heart perfusion is reported in our previous study [13]. In brief, cardiopulmonary bypass was instituted via double atrial and aortic cannulation. During antegrade heart perfusion, the pressure of the proximal segment of the aortic root was maintained at 50-80 mmHg. During retrograde heart perfusion, a flow rate of 200-300 mL/min, together with a coronary sinus pressure of 45-60 mmHg was maintained.

In patients with fistula diameter less than1 cm and no aortic regurgitation, the aorta was unclamped throughout CPB. Antegrade heart perfusion was performed via the arterial cannula. During the procedure, the communication between aorta and chamber was obliterated using finger, followed in a Foley catheter, inflated with water. The techniques used to close the ruptured orifice and ventricular septal defect in the beating heart were similar to those using cardioplegic arrest.

In patients with fistula diameter greater than 1 cm, who also had aortic regurgitation, retrograde perfusion was used; the perfusate from CPB was delivered via a catheter, at a flow rates of 200-300 mL/min, and pressure of 45-60 mmHg. During retrograde perfusion, a Foley catheter was not required to stop flow in the shunt. An aortotomy was performed to close the aneurysms and repair the aortic valve, as is performed in arrested hearts. After the intracardiac procedures were completed, standard de-airing maneuvers were emplyed. Transesophageal echocardiography(TEE) evaluated the condition of the aortic valve during the procedures. Perfusion adequacy was monitored by electrocardiography, blood returning from the coronary ostia, and myocardial color. After completion of the intracardiac manipulation, the nasopharyngeal temperature was raised to 36-37°C, and weaning from CPB was initiated.

Aortic valve replacement and ventricular septal defect repair using this technique have been explained in our previous study [13]. Aortic valvuloplasty, right ventricular outflow construction, and radical resection of subaortic stenosis were performed as in arrested hears.

## Results

There were no early or late deaths. All patients were in New York Heart Association (NYHA) functional class I at late follow-up (range, 0.5-1 year). Mild-to-moderate aortic valve regurgitation was observed in one patient. There was no recurrence of left-to-right shunt from the ruptured aneurysms of the sinus of Valsalva or aortic regurgitation due to sinus of Valsalva distortion.

## Discussion

Mean survival rate of patients with ruptured aneurysms of the sinus of Valsalva is 1-2 years. Congestive heart failure is known to be the main cause of death [20]. Hence, the presence of left-to-right shunt caused by ruptured aneurysms of the sinus of Valsalva is an indication for surgical intervention. Presence of congestive heart failure suggests a large shunt, and warrants urgent operation. Conventional myocardial protection strategies may further impair left ventricular function due to ischemia-reperfusion injury. This is particularly so in patients with additional cardiac abnormalities that are concomitantly corrected with ruptured aneurysms of the sinus of Valsalva. Therefore, better myocardial protection strategies are very important in patients with congestive heart failure or coexistent lesions. On-pump beating-heart surgery is characterized by avoidance of ischemia-reperfusion injury and cardioplegia [13-19]. It may benefit to patients with ruptured aneurysms of the sinus of Valsalva. In our study, all operations were performed on a beating heart. There was no postoperative low cardiac output syndrome or serious arrhythmias.

During on-pump beating-heart surgery, perfusion road was determined on the basis of coexistence of aortic regurgitation or diameter of the fistula. In patients with aortic regurgitation or in whom the diameter of the fistula was >1 cm, retrograde perfusion was used. In other patients, antegrade perfusion was used. In our study, retrograde perfusion was used in nine patients, and antegrade perfusion was used in twenty two patients. During retrograde perfusion, the shunt was obliterated after the aorta was cross-clamped, and a bloodless field was obtained. However, during antegrade perfusion, to obtain a bloodless field, a Foley's catheter was inflated with water across the shunt temporarily. Patients with combined lesions of ventricular septal defect and aortic regurgitation were easily treated using on-pump beating-heart technique. TEE was used to evaluate the aortic valve during the procedures and to guide de-airing. Antegrade perfusion is more physiological than retrograde perfusion, and retrograde perfusion does not provide adequate protection to the hypertrophied heart in an empty and beating state [21]. Nevertheless, the postoperative recovery of patients using retrograde perfusion in the present and other studies [22-24] was excellent. Besides, surgical correction of ruptured aneurysms with antegrade perfusion is technically a little more demanding than with retrograde perfusion because of the higher pressure of the proximal segment of the aortic root and the large shunt. Thus, in the patients with a fistula diameter >1 cm, retrograde perfusion was used.

The surgical approach for closure of a ruptured aneurysm of the sinus of Valsalva was determined on the basis of its position and coexistent lesions. In patients without coexistent cardiac lesions, the fistulae were repaired within the chamber into which they ruptured. There are three approaches to repair ruptured aneurysms of the sinus of Valsalva: via the terminal chamber, via the aortic root, and via a combined approach. Transaortic repair of ruptured aneurysms of the sinus of Valsalva may cause postoperative aortic regurgitation as a result of progressive distortion of the aortic sinus geometry [25]. However, others propose that a combined approach offers more advantages. Thus meticulous closure of the fistula as well as effective myocardial protection via cardioplegic arrest may decreased the incidence of late aortic insufficiency [4,26,27]. However, with on-pump beating-heart surgery, the advantages offered by the combined approach were achieved using TEE. The condition of the aortic valve could be evaluated under near-physiological conditions. In patients without associated aortic regurgitation, repairs were performed via the cardiac chamber into which rupture occurred. In patients with associated aortic regurgitation, closure of the aneurysms and aortic valve surgery were performed via the aortotomy approach. In patients with associated ventricular septal defect, a right ventricular incision was required.

The limitations of the study are (1) this was a cohort study performed at one institution, without a control group of patients. Other clinics might have different experiences, (2) this was a retrospective study and the different results among patients could represent preferences of the surgeons and anesthesiologists. This could have influenced the morbidity and long-term functional status, and (3) there was a relatively short follow-up period. Longer follow-up period may reveal the durability of this procedure performed on the beating heart.

## Conclusion

Beating heart on pump allows adequate examination of the aortic lesion under near-physiologic conditions, allows decrease in ischemia-reperfusion injury and potentially decrease the risk of serious or fatal rhythm disturbances. On-pump beating-heart technique for repair of ruptured aneurysm of sinus of Valsalva is feasible and promising. Antegrade heart perfusion is suitable for patients with a fistula diameter <1 cm and no aortic regurgitation, and retrograde perfusion is suitable for the rest.

## Competing interests

The authors declare that they have no competing interests.

## Authors' contributions

AM conceived the study, participated in its design and coordination, and performed the statistical analysis. HL conceived the study, and participated in its design and coordination. Both authors have read and approved the final manuscript.
